# Complete Genome Sequences of Novel Reassortant H1N2 Swine Influenza Viruses Isolated from Pigs in the Republic of Korea

**DOI:** 10.1128/genomeA.00552-13

**Published:** 2013-08-08

**Authors:** Philippe Noriel Q. Pascua, Gyo-Jin Lim, Hyeok-il Kwon, Young-il Kim, Eun-Ha Kim, Su-Jin Park, Se Mi Kim, Arun G. Decano, Eun-Ji Choi, Hae-Young Jeon, Chul-Joong Kim, Young-Ki Choi

**Affiliations:** College of Medicine and Medical Research Institute, Chungbuk National University, Heungduk-ku, Cheongju, Republic of Koreaa; College of Veterinary Medicine, Chungnam National University, DaeJeon, Republic of Koreab

## Abstract

Novel reassortant swine H1N2 influenza viruses were isolated from pigs in a commercial slaughterhouse in the Republic of Korea. Genome sequence analyses revealed that these isolates contain segments from Eurasian avian-like swine (hemagglutinin [HA]), Korean swine H1N2 (neuraminidase [NA]), and North American H3N2pM-like (remaining genes) viruses. Further characterization is needed to gauge the potential threats of these viruses to public health.

## GENOME ANNOUNCEMENT

Swine influenza viruses (SIVs) of the H1N1, H1N2, and H3N2 subtypes remain endemic in major swine populations worldwide (1). In the Korean Peninsula, North American-lineage triple-reassortant SIVs (bearing a combination of human-like, classical swine-like, and avian-like segments) have been exclusively detected for at least a decade (2). Predominated by H1N2 strains, these viruses have evolved independently from their North American SIV counterparts, forming their own lineage in Korean swine herds (3). The novel swine-origin A(H1N1) pdm09 virus was also found in Korean pigs (4) and reassorted with a preexisting Korean H1N2 SIV (5). More recently, we (Pascua et al., submitted for publication) and others (6) also isolated triple-reassortant H3N2 SIVs containing the pandemic M segment (H3N2pM), which was first identified in the United States (7). The establishment of these H3N2pM-like viruses in Korea would certainly complicate further the genetic diversity of SIVs in domestic pigs.

In April 2013, 12 H1N2 SIVs were isolated from monolayers of Madin-Darby canine kidney cells infected with supernatants of homogenized swine lung tissue collected in a commercial slaughterhouse located in Chungcheongbuk-do, Republic of Korea, which caters to various swine farms in the Chungcheong provinces. The whole-genome sequences were characterized and generated by reverse transcription PCR using influenza-specific primers as previously described (4), followed by sequencing with an ABI 373xl DNA sequencer (Applied Biosystems). Sequences were analyzed and assembled using DNAStar version 5.0.

Genetic analysis revealed that all isolates are highly identical to each other (99 to 100% homology), suggesting that they came from the same farm. The viral genome, represented by A/swine/Korea/CY0423-12/2013(H1N2), consists of 8 single-stranded RNA segments: polymerase basic 2 (PB2), PB1, polymerase (PA), hemagglutinin (HA), nucleoprotein (NP), neuraminidase (NA), matrix (M), and nonstructural protein (NS), with corresponding nucleotide lengths of 2,341, 2,341, 2,233, 1,778, 1,565, 1,466, 1,027, and 890. Encoded viral proteins and amino acid lengths include PB2 (759), PB1 (757), PB1-F2 (90), PA (716), PA-X (232), HA (566), NP (498), NA (469), M1 (252), M2 (97), NS1 (219), and NS2 (121). Phylogenetic analyses revealed that the HA and NA genes belong to Eurasian avian-like H1 SIVs circulating in Hong Kong and preexisting Korean H1N2 SIVs, respectively. The internal segments clustered together with contemporary North American triple-reassortant H3N2pM-like SIVs, including the Korean isolates in 2012 (6). The mature HA proteins displayed no polybasic amino acid (aa) residues in the cleavage site. However, residues 187D and 222E (H1 numbering) at the receptor binding pocket of the HA protein imply that these viruses might preferentially bind to α-2,6–linked sialosides (8). Except for the V27A and S31N substitutions in M2, no other remarkable markers associated with transmissibility, virulence, or resistance were present in the other viral proteins. Overall, we report for the first time the isolation of a novel genotype of H1N2 SIV with a gene constellation derived from multiple viral ancestors, most notably from Eurasian avian-like SIVs. Given that pigs can facilitate the generation of variant viruses with pandemic potential, our findings warrant a pathobiological assessment of these viruses to determine their potential threat to public health.

### Nucleotide sequence accession numbers.

The genome sequence of A/swine/Korea/CY0423-12/2013(H1N2) has been deposited in GenBank under accession no. KF142492 to KF142499.
